# DNA concentrations in amniotic fluid according to gestational age and fetal sex: data from 2573 samples

**DOI:** 10.1007/s00404-024-07698-6

**Published:** 2024-08-29

**Authors:** Yoel Gofin, Ran Svirsky, Dana Lavi Ben Atav, Meytal Liberman, Tamar Tenne, Sharon Perlman, Rivka Sukenik-Halevy

**Affiliations:** 1 Genetics Institute, Meir Medical Center, Kfar Saba, Israel; 2 School of Medicine, Faculty of Medical and Health Sciences, Tel Aviv University, Tel Aviv, Israel; 3 Genetic Unit, Department of Obstetrics and Gynecology, Samson Assuta Ashdod University Hospital, Ashdod, Israel; 4 Faculty of Health Sciences, Ben-Gurion University of the Negev, Be’er Sheva, Israel; 5 Rabin Medical Center, Ultrasound Unit, Helen Schneider Women’s Hospital, Petah Tikva, Israel

**Keywords:** Amniocentesis, Prenatal genetic testing, DNA concentration, Fetal sex, Gestational age

## Abstract

**Purpose:**

In some cases of prenatal genetic testing, an ample amount of fetal DNA is needed, to allow for parallel testing (conducting several genetic tests simultaneously). This study investigated the association between amniotic fluid DNA concentration and various factors. We aimed to define the required amount of amniotic fluid to be extracted in amniocentesis, to allow parallel testing throughout gestational weeks.

**Methods:**

DNA concentration was analyzed from amniocentesis samples taken during the years 2016–2022. Sex association was also analyzed in postnatal whole blood samples from a separate cohort. Theoretical minimum volume of amniotic fluid needed to ensure enough DNA for chromosomal microarray analysis and exome sequencing was calculated.

**Results:**

We focused our analysis on 2573 samples, which were taken during weeks 17–23 and 30–35. DNA concentrations increased from weeks 17 to 21, with relatively stable concentrations thereafter. Significantly higher DNA concentrations were seen in pregnancies of female fetuses. DNA concentrations in postnatal whole blood samples did not show this association. Across most weeks, the volume needed to extract 2 µg of DNA from 95% of the samples was about 34 ml.

**Conclusion:**

DNA concentrations in amniotic fluid vary according to gestational age and are higher in pregnancies of female fetuses. This should be considered when determining the volume of fluid extracted and the timing of amniocentesis, with greater volumes needed in earlier stages of pregnancy.

## What does this study add to the clinical work

**Table:** 

This is the first large scale description of DNA concentrations in amniotic fluid throughout gestation. The study shows that early amniocentesis requires extraction of greater volume of fluid, and that pregnancies with female fetuses have higher DNA concentrations.	

## Introduction

Amniocentesis is an invasive procedure performed for several indications, primarily for genetic work-up. DNA extracted from amniotic fluid cells is used for genetic tests, such as chromosomal microarray analysis (CMA) and Next Generation Sequencing (NGS) platforms such as gene panels and exome sequencing [1–5]. Testing is typically conducted sequentially, utilizing substantial amounts of DNA extracted from amniotic cell cultures. However, certain situations may warrant conducting CMA and NGS, simultaneously. This requires sufficient DNA extracted directly from amniotic fluid. Hence, determining the factors associated with amniotic fluid DNA concentrations is relevant when deciding on the volume of fluid that should be extracted during amniocentesis.

Previous studies, reporting a relatively small scale, showed some variability in amniotic fluid DNA concentrations, with higher concentrations in the later weeks of gestation [6–8].

The main indications for amniocentesis in Israel are advanced maternal age, abnormal maternal biochemical serum screening, congenital malformations and molecular diagnosis of genetic diseases.

Amniocentesis is usually conducted during 17–23 weeks of gestation. However, in certain scenarios, it may be delayed until later in pregnancy [9], typically after 30 weeks, to mitigate the risk of extreme premature labor. Nevertheless, testing can be performed at any gestational age based on clinical considerations. Some indications, such as polyhydramnios, overgrowth, and intrauterine growth restriction, typically present in the later stages of pregnancy. Indications for late testing are expected to be more prevalent with the introduction of the third trimester scan by professional societies, such as the International Society of Ultrasound in Obstetrics and Gynecology (ISUOG) [10]. In these cases, amniocentesis may be performed very late in gestation, potentially necessitating parallel testing.

In many labs, requirements for genetic testing include a minimum of 200 ng of DNA for CMA and 1–2 µg for exome sequencing.

The aim of this study was to discover parameters associated with DNA concentrations using a database of amniotic fluid samples.

## Methods

DNA concentrations in amniotic fluid samples obtained at our institution were analyzed. Inclusion criteria were all amniocentesis samples obtained from September 2016 to September 2022. Exclusion criteria were the use of ethanol precipitation to concentrate DNA and samples from cases with missing data. We examined the association between DNA concentration and gestational age, as well as maternal age, fetal sex (as determined by genetic testing), and indication for testing.

As a comparison group, we analyzed DNA concentrations in our internal database of postnatal whole blood samples to assess any potential associations between DNA concentrations and patient sex in a different biological medium. Blood samples were taken from a fixed volume of 400 µl.

We calculated the theoretical minimum volume needed to ensure enough DNA for parallel CMA and exome testing (2 µg of DNA) based on the average amniotic fluid DNA concentrations for each week. Additionally, we calculated the minimum volume required for parallel testing in a given week, ensuring that 95% of the samples would allow this testing.

### Statistical analysis

Two-sided, type 2 student t-tests were used to measure the association between DNA concentrations at different gestational weeks and the study parameters including fetal sex, indication for testing and maternal age. A P-value of less than 0.05 was considered statistically significant.

Second order linear regression was used to describe the trend between DNA concentration and gestational age. Python version 3.5.1 was used to analyze the data and to create the plots using the following libraries: numpy, scipy.stats, matplotlib and pandas.

## Ethics

The study was approved by the Meir Medical Center Institutional Review Board (0169–21-MMC). Patient informed consent was not required.

## Results

From September 2016 to September 2022, DNA was extracted from 2733 amniocentesis procedures. After excluding 108 samples due to missing data and/or use of ethanol precipitation to concentrate DNA, 2625 were available for analysis. The analysis was performed only for weeks in which there were more than 10 samples: weeks 17–23 and 30–35, for a total of 2573 samples. The characteristics of the study cohort are presented in Table 1.

**Table 1:** Characteristics of the study cohort of 2573 samples analyzed

Variable	*N* (%)
Fetal sex*	
Female	1226 (47.6)
Male	1287 (50)
Not documented	60 (2.3)
Indication	
Advanced maternal age	1441 (56)
Congenital malformations	307 (11.9)
No indication	395 (15.4)
Abnormal biochemical survey^†^/non-invasive prenatal screening	251 (9.8)
Nuchal translucency ≥ 3 mm	49 (1.9)
Abnormal genetic test result in a previous pregnancy	33 (1.3)
Polyhydramnios	20 (0.8)
Testing for genetic syndromes through the national carrier screening program or for known familial variants	17 (0.7)
No indication documented	60 (2.3)
Maternal age, years (median, range)	36, 18–51
Under 25, *n* (%)	42 (1.6)
25–29, *n* (%)	246 (9.6)
30–34, *n* (%)	602 (23.4)
35–39, *n* (%)	1201 (46.7)
≥ 40, *n* (%)	422 (16.4)
Not documented, *n* (%)	60 (2.3)

*As ascertained by fetal CMA

^†^Down syndrome risk of 1:380 or higher according to biochemical screening

## Analysis according to gestational age

**Fig. 1:**
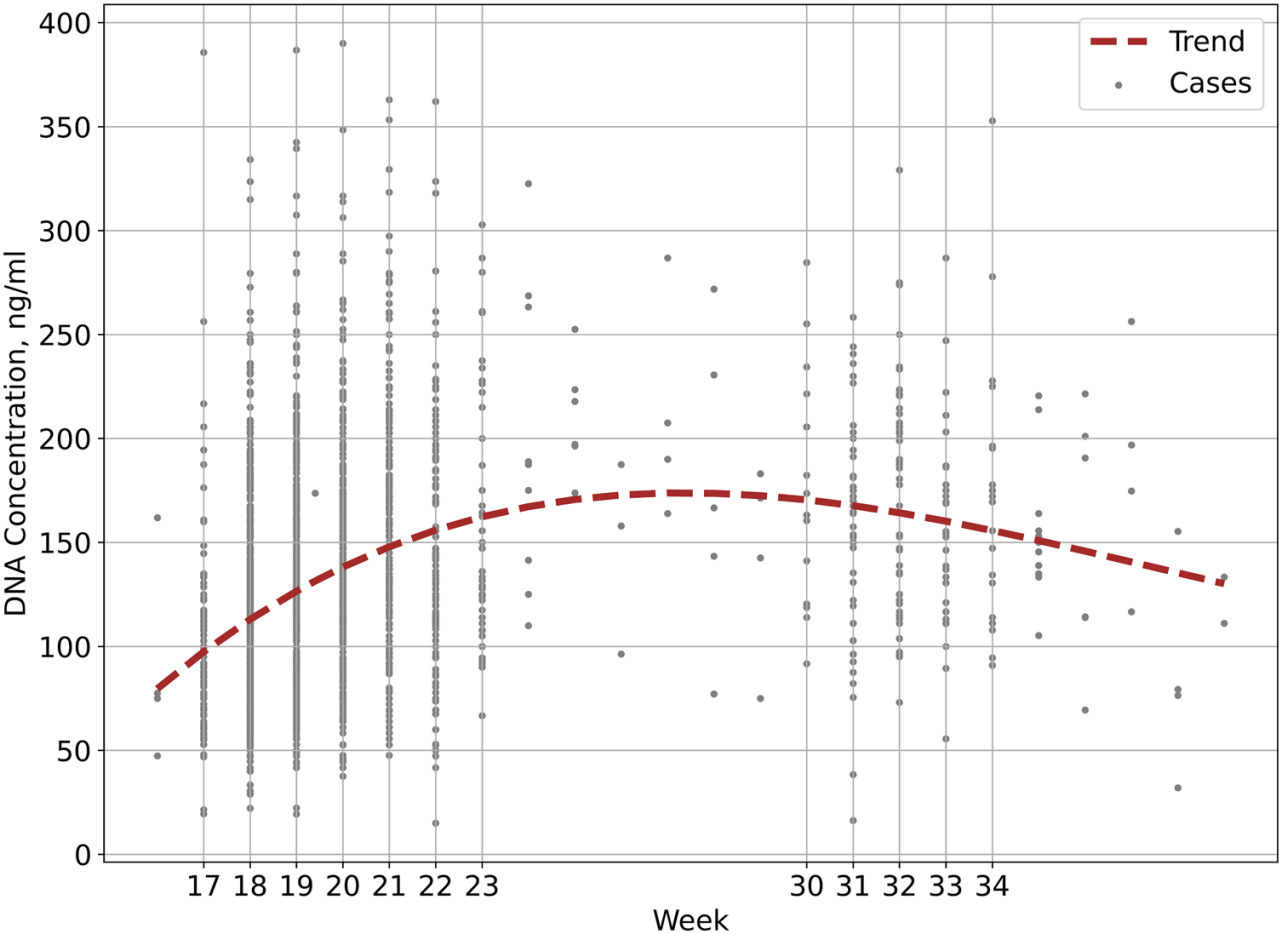
DNA concentration in 1 ml of amniotic fluid, according to gestational age. Second order linear regression was used

The number of cases and the DNA concentrations at each gestational week are presented in Table 2. The distribution of concentrations throughout gestation is depicted in Fig. 1 (the figure also includes samples from weeks that were not analyzed).

**Table 2:** DNA concentrations at each gestational week analyzed

Gestational week	No. of samples	DNA concentration in amniotic fluid, average, ng/ml (SD)	Volume (ml) needed for 2 µg DNA, based on average concentration	Minimum volume (ml) for which 95% of samples have ≥ 2 µg DNA
17	119	96.75 (46.37)	20.7	37.9
18	802	113.15 (43.51)	17.7	34.3
19	702	125.56 (47.88)	15.9	30.8
20	416	138.44 (51.25)	14.4	30.8
21	195	155.51 (60.84)	12.9	31.2
22	123	145.25 (61.77)	13.8	38.1
23	45	156.87 (61.32)	12.7	22.2
24*	9	198.03 (68.10)	10.1	
25*	6	210.22 (24.86)	9.5	
26*	3	147.23 (37.99)	13.6	
27*	4	212.04 (45.86)	9.4	
28*	5	177.9 (67.95)	11.2	
29*	4	143.02 (41.94)	14.0	
30	15	178.14 (54.30)	11.2	17.6
31	47	155.68 (52.98)	12.8	26.7
32	49	168.81 (55.81)	11.8	21
33	29	154.80 (48.45)	12.9	22.3
34	20	167.31 (65.16)	12.0	22
35	11	155.89 (32.43)	12.8	15
36*	6	151.77 (55.39)	13.2	
37*	4	186.14 (49.97)	10.7	
38*	4	85.74 (44.35)	23.3	
39*	2	122.22 (11.11)	16.4	

*Weeks not included in analysis due to small number of samples

*SD* standard deviation

**Fig. 2:**
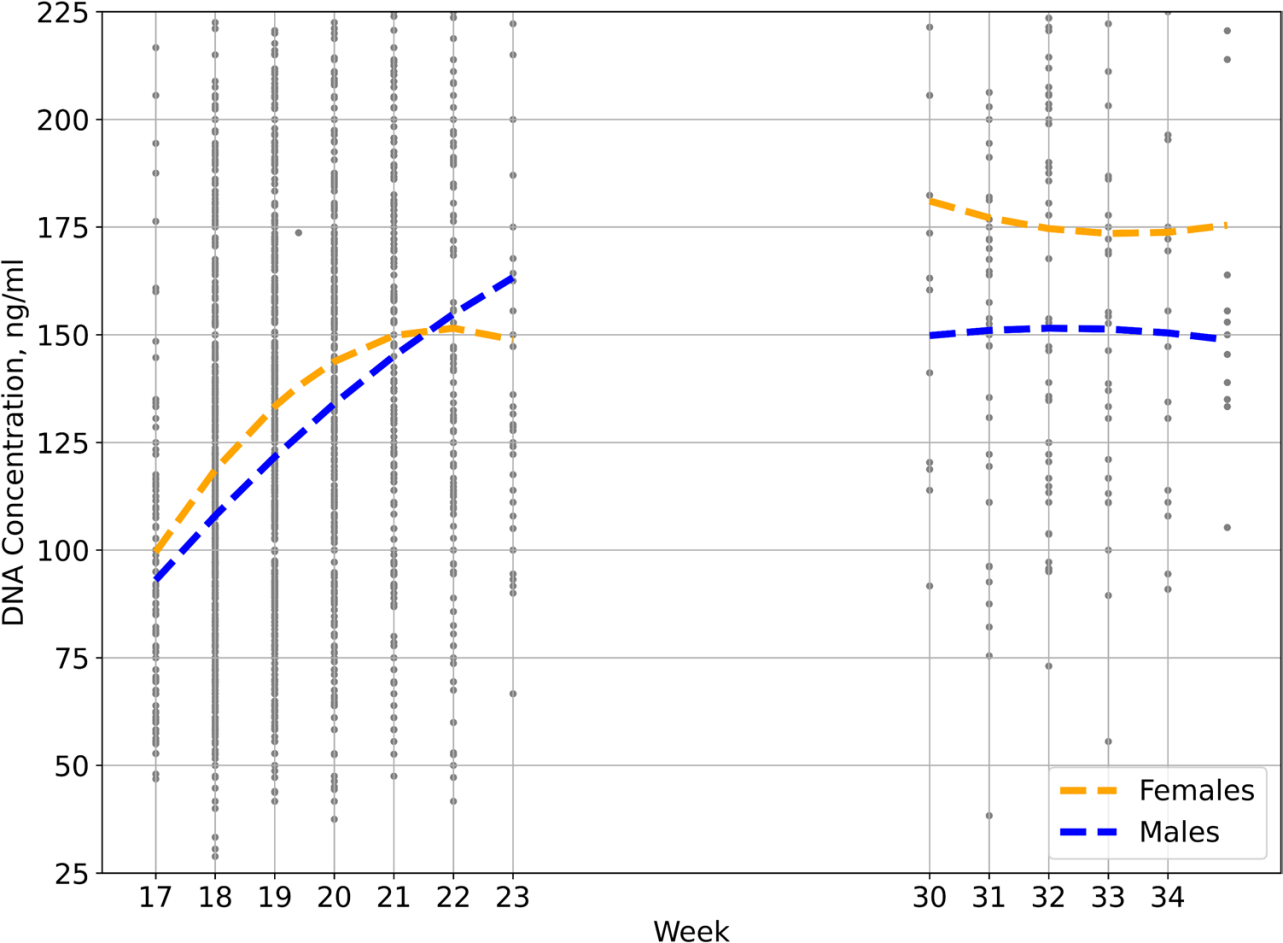
DNA concentrations in 1 ml of amniotic fluid, according to gestational age and fetal sex. Second order linear regression was used. Data for weeks 24–30 are not shown due to a small number of samples

Most samples (2402, 93.3%) were taken during weeks 17–23. During this period, concentrations steadily increased, and approximately doubled between 17 weeks and 23 (Fig. 2). After this period, levels were relatively stable. In most weeks analyzed, the minimum volume to ensure sufficient DNA for conducting CMA and exome testing in parallel (2 µg), based on the average DNA concentration was calculated as 18 ml. In the early weeks of testing, a relatively large portion of samples had a DNA concentration that required larger volumes of amniotic fluid to reach 2 µg of DNA. In weeks 17–22, 38 ml of amniotic fluid was needed to secure enough DNA in 95% of the samples (in most weeks, 34 ml was sufficient). However, in the later weeks of testing, the threshold was lower, and 22 ml was sufficient for 95% of samples in most weeks (a few samples in week 31 were low concentration outliers).

## Analysis in terms of fetal sex

The DNA concentration in pregnancies of female fetuses was higher than in males (Table 3). Statistical significance was shown in weeks with high numbers of samples (18–20). A similar trend for higher concentrations was observed in all other gestational weeks, except for week 22. (Table 3, Fig. 2).

**Table 3:** DNA concentrations according to fetal sex

Gestational week	DNA concentrations, ng/ml (number of cases)		Male:Female ratio	*P*-value
	Males	Females		
17	91.4 (55)	102.5 (62)	0.89	0.198
18	108.8 (400)	118 (387)	0.92	**0.003**
19	119.6 (348)	131.9 (338)	0.90	**0.001**
20	132.7 (205)	144.6 (206)	0.92	**0.019**
21	150.3 (110)	162 (84)	0.93	0.188
22	153.3 (69)	132.4 (52)	1.16	0.059
23	158.3 (21)	161 (21)	0.98	0.893
30	151.9 (7)	201.1 (8)	0.76	0.09
31	149.8 (19)	161.8 (27)	0.93	0.461
32	151.6 (26)	188.3 (23)	0.81	**0.021**
33	150.6 (21)	165.7 (8)	0.91	0.471
34	155 (9)	177.4 (11)	0.87	0.47
35	145.4 (6)	168.5 (5)	0.86	0.355

Statistically significant values (*P* < 0.05) are written in bold

The analysis of 449 postnatal whole blood samples (243 male, 206 female), did not show significant differences in DNA concentrations based on sex (*p* = 0.89).

For each gestational week, the DNA concentration for each amniocentesis indication was compared to all other indications, combined. No significant differences were seen for any indication. Additionally, there were no discernible differences noted according to maternal age.

## Discussion

To the best of our knowledge, this report represents the first large-scale study to report DNA concentrations in amniotic fluid over the entire course of pregnancy, when amniocentesis is feasible. The data show that DNA concentrations increase between 17 and 22 weeks of gestation, with levels relatively stable thereafter. A similar trend was observed in a smaller study of 90 samples, between weeks 13 and 34 [8]. A smaller study of 15 samples showed relatively similar concentrations and higher concentrations in later weeks but with high variability [7].

Our data show that DNA concentrations did not decrease in late gestation. This finding indicates that amniocentesis following late sonographic findings (third trimester scan) would still allow for genetic testing, with similar volume requirements of extracted fluid.

When considering the formal requirements for DNA amounts and concentrations, early amniocentesis appears to pose a greater risk of insufficient DNA to allow parallel direct testing.

However, based on our laboratory’s experience, instances of insufficient DNA are relatively rare, and testing often proves successful even under less-than-optimal conditions. Also, DNA quantity and quality may depend on lab specific parameters such as extraction protocols and equipment used, hence volume requirements may be different between labs. In our lab, a volume of 34–38 ml of amniotic fluid appears necessary to ensure sufficient DNA for parallel direct testing in approximately 95% of cases, establishing a robust standard. A laboratory can define a lower threshold for DNA amount and allow for lower fluid volume requirements, providing the success rate is kept at a satisfactory level.

The most striking finding in our study is that amniotic fluid from female fetuses has higher concentrations of DNA. This difference was not seen in postnatal whole blood samples. Therefore, this phenomenon cannot be explained by the differences in the sex chromosomes between female and male genomes.

The DNA extracted from amniotic fluid is derived from exfoliated cells from the fetal skin, urinary and gastrointestinal tracts, and respiratory system [11], and the DNA concentration depends on both the number of cells present in the amniotic fluid and on the amniotic fluid volume.

One explanation may be a physiological difference between fetal sexes in amniotic fluid volume. A study that assessed almost 300 pregnancies [12] with idiopathic polyhydramnios reported that 73% of cases involved male fetuses. It has also been suggested that the amniotic fluid volume may be associated with fetal biometry [13–15]. Postnatal growth charts, as well as prenatal nomograms show that males are generally larger than females [16–18]. Consequently, the amniotic fluid volume of female pregnancies may be lower, leading to higher DNA concentrations.

Another factor that might contribute to the sex difference found in our study might be the differences in the anatomy of the genitalia between the sexes, with the female urinary system shedding more cells from the urinary tract to the amniotic fluid. In addition, urine production increases significantly after week 20 [19], contributing to the difference between the sexes in the later weeks of gestation.

The nadir in DNA concentration was observed only in the curve of female gestations (Fig. 2). This interesting phenomenon may be linked to physiological differences between male and female fetuses and the patterns of urinary tract cell exfoliation into the amniotic fluid. Further research may be required to explain this observation.

This study did not detect an association between DNA concentrations and the indication for testing. It is possible that an association might become apparent if larger sample sizes for all indications are evaluated.

As expected, we did not find an association between DNA concentration and maternal age, supporting the hypothesis that fetal factors play a predominant role in determining amniotic fluid DNA concentrations. Our data do not point to a single predictive factor for low DNA concentrations, other than the timing of amniocentesis.

This study had several limitations. The diabetes status of women, which can cause an increased amount of amniotic fluid, was not included in the analysis. In addition, the data did not allow for factoring specific congenital anomalies in the analysis (for example, urinary system anomalies could have affected the amount of amniotic fluid). Lastly, the results of the CMA (abnormal vs. normal) were not considered when analyzing DNA concentrations according to the factors mentioned.

In conclusion, DNA concentrations in amniotic fluid vary across gestational ages, demonstrating an increase between 17 and 21 weeks, followed by relatively stable levels thereafter. Extraction of 34–38 ml of amniotic fluid should be sufficient for parallel direct testing at all gestational ages. Lower volumes should be sufficient in later weeks, especially in the late phase of 30 weeks and onwards. Female fetuses exhibit higher DNA concentrations in amniotic fluid. Further studies are required to determine whether additional factors contribute to differences in DNA concentrations, such as fetal size or ultrasound abnormalities.

## Data Availability

The data that support the findings of this study are not openly available due to reasons of sensitivity and are available from the corresponding author upon reasonable request.
